# 4575 Implementing a Workflow Management Tool for Clinical Trials

**DOI:** 10.1017/cts.2020.132

**Published:** 2020-07-29

**Authors:** Laura Nelle Hanson, Jennifer Weis, Sasa Andrijasevic, Sharon Elcombe, Rachel Hardtke, Andrea Kukla, Linda Sanders

**Affiliations:** 1 Mayo Clinic

## Abstract

OBJECTIVES/GOALS: A workflow management tool is essential in order to help support consistent processes with transparency in next steps of the study process. Prior to this tool, staff has relied upon extensive training and coaching on the study process. While resources and guidelines exist, it requires additional time for staff to identify these resources and allows for confusion and rework. Implementation of a systematic workflow management tool was identified as a critical need in order to support streamlined processes, improve transparency and support business continuity, and to accelerate the study process. METHODS/STUDY POPULATION: This effort was undertaken as part of the Protocol Lifecycle Management effort to implement a comprehensive clinical trial management system for clinical research studies. Mayo Clinic has designed a workflow management tool within the Velos eResearch system. The workflow manager is dynamic and will present specific activities based on the study design and responses to data entered on the ad hoc forms. A Workflow Build group contributed to the design of the workflow in order to reflect appropriate, current operational processes. The workflow was vetted and validated with research teams. In addition to designing activities, planned dates and target timelines were established for relevant workflows to help promote transparency in the study start-up timelines and allow study staff to identify overdue activities. Study status controls were designed in the workflow to protect study staff from inadvertently changing the status until appropriate activities are complete. RESULTS/ANTICIPATED RESULTS: A dynamic workflow has been designed and implemented in the Velos eResearch system to support Mayo Clinic research sites. This system will be implemented February 24, 2020 to all consenting studies. DISCUSSION/SIGNIFICANCE OF IMPACT: The implementation of this workflow management tool is critical to help support research operations in a large, academic medical center. Benefits to implementation are expected to include improved transparency in the study status and next steps, reductions in rework due to confusion in next steps, better understanding from new staff in the appropriate study process, and improved timelines for study start-up. As we prepare for the implementation of the Velos eResearch system at Mayo Clinic, the workflow management tool has been identified in training sessions as a positive benefit.

